# Study on the Biodegradation of Poly(Butylene Succinate)/Wheat Bran Biocomposites

**DOI:** 10.3390/ma16216843

**Published:** 2023-10-25

**Authors:** Emil Sasimowski, Łukasz Majewski, Marta Grochowicz

**Affiliations:** 1 Department of Technology and Polymer Processing, Faculty of Mechanical Engineering, Lublin University of Technology, Nadbystrzycka 36, 20-618 Lublin, Poland; l.majewski@pollub.pl; 2 Department of Polymer Chemistry, Institute of Chemical Sciences, Faculty of Chemistry, Maria Curie-Sklodowska University, Gliniana 33, 20-614 Lublin, Poland; mgrochowicz@umcs.pl

**Keywords:** biocomposite, biodegradation, biofiller, agro-waste materials, agro-flour filler, natural filler, thermal resistance, molecular mass, lignocellulosic materials, biopolymer, composites

## Abstract

This paper presents the results of a study investigating the biodegradation of poly(butylene succinate) (PBS)/wheat bran (WB) biocomposites. Injection mouldings were subjected to biodegradation in compost-filled bioreactors under controlled humidity and temperature conditions. The effects of composting time (14, 42 and 70 days) and WB mass content (10%, 30% and 50% wt.) on the structural and thermal properties of the samples were investigated. Measurements were made by infrared spectral analysis, scanning electron microscopy, differential scanning calorimetry, thermogravimetric analysis, and gel permeation chromatography. Results demonstrated that both the thermal and structural properties of the samples depended greatly on the biodegradation time. Specifically, their crystallinity degree increased significantly while molecular mass sharply decreased with biodegradation time, whereas their thermal resistance only showed a slight increase. This resulted from enzymatic hydrolysis that led to the breakdown of ester bonds in polymer chains. It was also found that a higher WB content led to a higher mass loss in the biocomposite samples during biodegradation and affected their post-biodegradation properties. A higher bran content increased the degree of crystallinity of the biocomposite samples but reduced their thermal resistance and molecular mass.

## 1. Introduction

Indiscriminate use of polymeric materials of petrochemical origin in the manufacture of products, especially those intended for short-term use, poses a serious threat to the environment due to the resulting waste products [1,2,3]. According to the OECD Global Plastic Outlook, 353 million tonnes of plastic waste was produced worldwide in 2019 [4]. Given the growing awareness of this threat, as well as the legal restrictions imposed on the use of such materials, an increasing interest in polymeric biodegradable materials has been observed for several years [5,6,7,8,9,10,11]. The annual production of biodegradable polymers in 2023 is expected to amount to 0.7 million tonnes [12]. Therefore, the problem of developing new fully biodegradable plastics, i.e., biopolymers and their composites, has been investigated in numerous studies [13,14,15,16,17,18,19].

Biopolymers are materials capable of undergoing biodegradation under environmental conditions with the participation of microorganisms [20,21,22]. It should be emphasized that biodegradable polymers indicate different course and rate of degradation, which depends mainly on the chemical structure of macromolecules, which in turn determines water solubility, thermal resistance, chemical activity and resistance to enzymes. The relationship between the chemical structure of polymers and their degradability depending on various external factors has been demonstrated in many studies [23,24,25,26,27,28,29,30]. In addition to biodegradation by microorganisms, other types of degradation can also be distinguished, including thermal degradation at elevated temperatures, mechanical degradation caused by prolonged stress, oxidative degradation in oxygen-containing atmospheres, photodegradation caused by light radiation, hydrolytic degradation caused by high humidity, corrosion caused by chemical activity and degradation caused by high-energy electromagnetic radiation (e.g., UV) [22,31,32,33,34]. The interaction of these factors leads to irreversible changes in the structure of polymeric materials, such as macromolecular chain shortening and crystalline phase proportion changes, which in turn causes changes in their mechanical and physical properties [35,36,37,38]. Increased stiffness and brittleness are usually followed by material fragmentation and, consequently, increased specific surface area as well as mass loss [39,40,41,42,43]. Nevertheless, the actual biodegradation process depends on the microbial activity causing the decomposition of biopolymers into simple substances such as water, carbon dioxide and inorganic compounds [32,33,41,44,45,46].

One of the most interesting biodegradable polymers is poly(butylene succinate) (PBS). This material exhibits attractive properties, such as compostability and biodegradability in both soil and water environment, high thermal and chemical resistance, as well as good mechanical properties [47,48,49,50,51,52,53].

Compared to other popular biodegradable plastics, PBS has many beneficial properties. The melting point of PBS (*T_m_* = 114 °C) is lower compared to PLA (*T_m_* = 165 °C), comparable to PBAT (*T_m_* = 110–115 °C), but higher than PCL for which *T_m_* = 60–65 °C [54]. PBS stands out with its excellent processability, which enables its processing by injection, extrusion and blow molding. Equipment and processing conditions similar to those for polyolefins are then used. The strength properties of PBS are definitely better than those of PLC and PBAT, while PLA is stiff and brittle [55]. The mechanical properties of PBS are similar to the most commonly used petrochemical plastics. The stiffness of PBS is intermediate between (LDPE) and (HDPE). The yield strength is comparable to (PP) but more than twice that of (LDPE). However, PBS has a relatively low biodegradation rate due to the high degree of crystallinity [54]. Therefore, it is desired to accelerate the degradation rate by using various types of fillers that will additonally reduce the price of the composition.

Numerous studies on PBS-matrix composites with plant-based fillers, such as jute fibres [56], silk [57], sisal [58], kenaf [59], bamboo [60], ground rice husks [61], wheat bran [62,63,64,65], wood shavings [66] and apple and grape pomace [19,67], can be found in the literature. Composites of PBS with mineral fillers such as chalk, talc [68], montmorillonite [69,70] and other aluminosilicates are also known. Composite biopolymers such as PBS with the aforementioned fillers are considered to be a promising alternative to traditional petrochemical plastics. Similarly to polycaprolactone and polylactide, PBS requires specified conditions for proper and rapid biodegradation, namely humidity, specific bacterial strains, as well as a suitable temperature and pH [41,51,71,72,73,74]. As a result, this material can be used for a long time under standard conditions and decomposes within a few months when subjected to industrial composting [51,53]. The hydrophilic nature of lignocellulosic fillers such as bran should also be taken into account when it comes to composite materials. Owing to their chemical structure, these fillers exhibit hydrophilic properties and are, thus, much less resistant to physical and chemical factors and microbial activity than PBS [75,76]. This can be a limiting factor, resulting in a shorter life of products made from biocomposites based on these fillers. Therefore, it is important to know the full characteristics of a biocomposite material, taking into account not only the processability and physical properties of this material but also the kinetics and course of the biodegradation process.

The authors of this paper have already conducted comprehensive studies on the PBS/wheat bran composite material. Previous studies investigated the processing of this composite material via twin screw extrusion [63], the performance of injection moulded samples of this material [64] and the resistance to aging and biodegradation [65] of the patented PBS/wheat bran composite [77]. It was shown that wheat bran content had a significant effect on the composite properties. This study is a continuation of that research. The aim of this study was to determine changes in the structural and thermal properties of this biocomposite following biodegradation by composting under controlled conditions. The effects of wheat bran mass content ranging from 10 to 50% wt. in biocomposite samples and composting times of 14, 42 and 70 days were studied under industrial composting conditions. There are known papers concerning the composting of PBS/inedible cereal flour blends [78] or the biodegradation of poly(butylene succinate-co-adipate)/wheat bran composite in seawater [79]. However, the available literature reports no studies on PBS/wheat bran biodegradation. The course and rate of the degradation process may be a key factor that will determine the technical potential of the tested composite and open up additional opportunities for its practical usage. Applications involving the encapsulation of substances and their controlled release in the environment require a knowledge of the degradation process in order to control its course and rate. The use of agricultural waste for the controlled release of fertilizers in agriculture is prospective and in line with a “zero waste” policy and the circular economy concept.

## 2. Material and Methods

### 2.1. Materials

The tested biocomposite was made of a poly(butylene succinate) matrix and a wheat bran biofiller. PBS in pellet form, with the trade name BioPBS FZ91 PB [80], was supplied by PTT MCC BIOCHEM Co., LTD. (Bangkok, Thailand). Wheat bran (WB), i.e., wheat grain husks in the form of thin flakes up to a few mm in size, was obtained from a mill near the city of Lublin (Poland).

### 2.2. Procedure for the Production of Biocomposite Materials

Polymer biocomposite pellets with 10%, 30% and 50% wt. bran contents were produced using an extrusion and pelleting processing line manufactured by Zamak Mercator (Skawina, Poland), equipped with the EHP-2 × 20 Sline co-rotating twin-screw extruder. The extruder’s screw speed was 125 min^−1^. Other extrusion and pelleting conditions were described in detail by Sasimowski et al. in [63]. Test samples were produced by injection moulding both from the biocomposite pellets and from pure PBS pellets. The injection moulding procedure and conditions were the same as in [64]. The shape and dimensions of the samples complied with the ISO 294-1:2017 standard [81]. The dog-bone-shaped specimens had a total length of 150 mm and a thickness of 4 mm. As a result, four types of materials were obtained, denoted by PBS, WB10 (with 10% WB), WB30 (with 30% WB) and WB50 (with 50% WB).

### 2.3. Biodegradation Experiment

The test stand for conducting biodegradation experiments consisted of bioreactors (polypropylene containers filled with compost) and a constant temperature and humidity chamber (Climabox LHS-150HC-II from Agencja Anticorr, Gdańsk, Poland). Biodegradation of the biocomposite samples was conducted under controlled conditions in compliance with ISO 20200:2015 [82]. The industrial compost for the experiment was obtained from a local waste management facility (Lublin, Poland).

Individual biocomposite samples were put in separate bioreactors located in a climate chamber with a temperature of 58 °C and a humidity of 60%. Water was replenished in the bioreactors, and the compost was homogenized at the intervals specified in the standard. After the specified biodegradation-composting time of 14, 42 and 70 days, the samples were extracted from the compost, washed and dried to achieve uniform mass. The post-biodegradation samples were denoted by adding to their names suffixes of 14; 42 or 70, corresponding to the composting time.

### 2.4. Materials Characterization

Infrared spectra (FTIR) were taken using the Tensor 27 spectrometer (Bruker, Germany) equipped with an attenuated total reflectance (ATR) module with a diamond crystal. The spectra were recorded from 600 to 4000 cm^−1^ with 32 scans per spectrum and a resolution of 4 cm^−1^. 

The morphology of the samples before and after specified composting times was examined with a scanning electron microscope (SEM) (FEI Quanta 3D FEG, FEI Company, Hillsboro, OR, USA) working at 5 kV. Prior to examination, the samples were coated with a thin layer of gold.

A thermogravimetric analysis was performed in synthetic air with the use of STA 449 F1 Jupiter (Netzsch, Günzbung, Germany) coupled with the FTIR TENSOR 27 spectrometer (Bruker, Mannheim, Germany). The measurement conditions were as follows: temperature range of 40–600 °C, heating rate of 10 °C/min, gas flow of 25 mL/min, sample mass of approx. 10 mg. The samples were analysed in Al_2_O_3_-opened crucibles.

Differential scanning calorimetry (DSC) was performed on DSC 204 F1 Phoenix (Netzsch, Günzbung, Germany) provided with the Netzsch Proteus software version 6, in accordance with the ISO 11357-1:2016 standard [83]. Each measurement was made in three cycles: heating from −150 °C to 140 °C with a heating rate of 10 K/min (heating I); cooling from 140 °C to −150 °C with a cooling rate of 10 °C/min; heating from −150 °C to 140 °C with a heating rate of 10 °C/min (heating II). The 10 mg mass samples were analysed in closed pierced aluminium pans in argon atmosphere with a flow rate of 25 mL/min. To ensure measurement accuracy, the temperature and heat flow rate were calibrated in the DSC apparatus using melting indium parameters (*T_m_* (onset) = 156.6 °C, Δ*H_f_* = 28.45 J/g). The temperature accuracy was 0.1 °C. Obtained thermograms were used to calculate parameters such as melting enthalpy (Δ*H_m_*), melting temperature (*T_m_*), crystallization temperature (*T_c_*), glass transition temperature (*T_g_*) and crystallinity degree (*X_c_*). The *T_g_* value was adopted as the inflection point of a DSC curve in the glass transition area. The *X_c_* parameter was calculated from the equation:

$$X_{c}=\frac{\Delta H}{(1-u)\times {\Delta H}_{100\%}}\times 100\%$$

where *u* is the weight fraction of WB in the composite sample, Δ*H* is the melting enthalpy, Δ*H*_100%_ is the melting enthalpy for 100% crystalline PBS, and its value is assumed to be 110.3 J/g [84].

The number average molecular mass (M_n_) and the weight average molecular mass (M_w_) were determined by gel permeation chromatography (GPC) using the Agilent 1200 modular HPLC series system (Agilent, Santa Clara, CA, USA) with a refractive index detector (RID). The system was equipped with two PLgel 5 µm MIXED-C (300 × 7.5 mm) columns connected in series. Calibration was performed on 12 polystyrene standards with the mass (M_n_) range of 474 g/mol–1,800,000 g/mol. Measurements were made at 35 °C. Chloroform (HPLC grade) with a flow rate of 0.6 mL/min was used as a mobile phase. Data were acquired using the ChemStation for LC program and analysed using the ChemStation 4.0 GPC Data Analysis Software. M_n_ and M_w_ were calculated using the retention volume values. The polydispersity index (PDI) was computed by dividing M_w_/M_n_ values. Before analysis, the samples were dissolved in chloroform at 37 °C and centrifuged for 10 min at 10,000 rpm in order to separate the filler from potential composting residues. After centrifugation, the solutions were filtered through a PTFE syringe filter with a pore size of 0.2 µm. The concentration of the analysed solutions was approximately 3 mg/mL. Each sample was analysed three times.

## 3. Results and Discussion

### 3.1. Mass Loss

**Figure 1:**
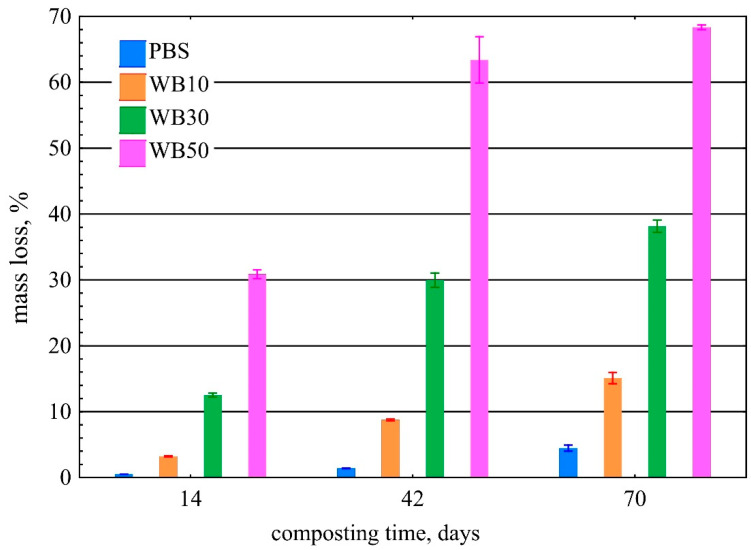
Mass loss in PBS and biocomposites versus composting time.

Results showed that mass loss in the composted samples was linear and depended on the biodegradation time (Figure 1). The samples made from PBS biodegraded very slowly, and their mass decreased on average by only 4.5% after 70 days. A similar slow degradation of PBS was also observed in a study by Puchalski et al. [53]. The mass loss of the wheat bran-containing samples was much greater and depended on the bran content. The addition of 10% wt. bran resulted in an over threefold increase in the mass loss of the samples, which averaged 15.1% after 70 days of biodegradation. This can be explained by bran’s high ability to absorb and retain water. As for its chemical structure, wheat bran is more easily enzymatically hydrolysed and is preferred by microorganisms [85], which has been confirmed by the SEM results presented in Section 3.4. Therefore, increasing the bran content in the biocomposite samples to 30% wt. resulted in a 38.1% loss in their mass after merely 70 days of biodegradation. As expected, the greatest mass loss occurred for the highest tested 50% wt. bran content in the composite material. After 70 days, the average mass loss in these samples was 68.3%. For this case, the considerable mass loss by the samples should further be associated with a higher rate of PBS matrix degradation. This was due to an increased surface area of the interaction between water and microorganisms on PBS following the swelling of water-absorbing bran. A similar increase in the biodegradation rate of PBS composites with other lignocellulosic fillers was observed in [47,51,84].

### 3.2. Chemical Structure

**Figure 2:**
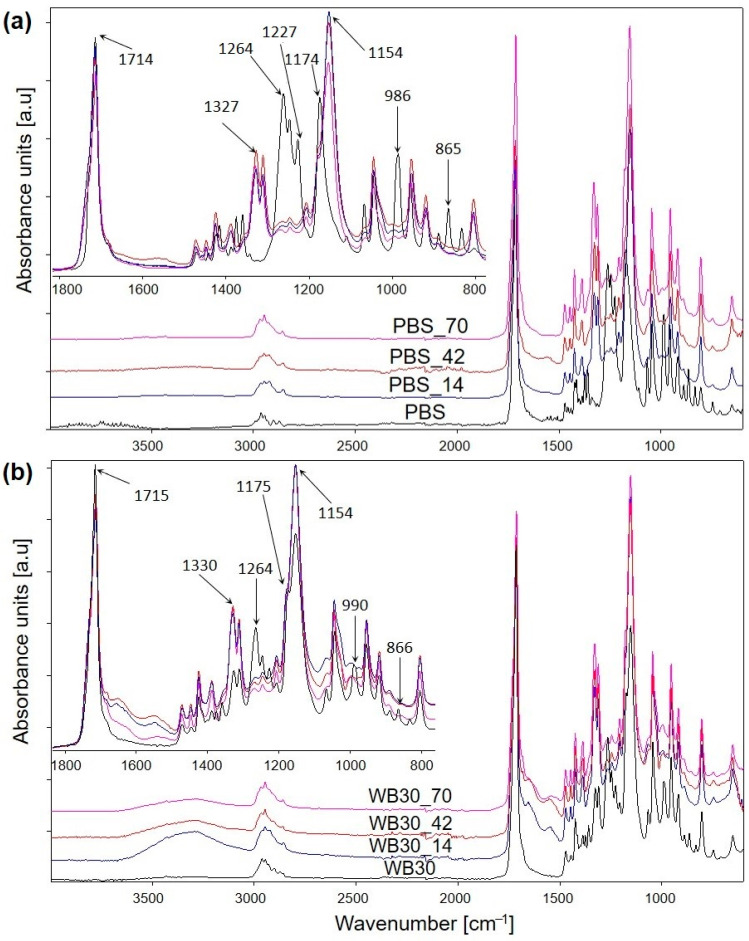
ATR-FTIR spectra of PBS (**a**) and biocomposite with 30% wt. bran (**b**) after different biodegradation times.

FTIR spectra were captured after 14, 42 and 70 days of biodegradation to determine changes in the chemical structure of the composted samples. Figure 2 shows the FTIR spectra taken for PBS and WB30 (containing 30% wt. of bran). The spectra of both PBS and biocomposite samples clearly differ before and after biodegradation. PBS is a polymer belonging to the group of aliphatic polyesters, and its chemical structure contains ester bonds that are susceptible to hydrolysis reactions. The biocomposite samples include wheat bran, which is a predominantly lignocellulosic material, but also contain proteins that are prone to enzymatic hydrolysis [51,85,86,87]. Structural changes were observed in the chemical structure of PBS and its composites just after 14 days of composting. The changes occurred mainly within ester groups. Compared to the initial samples, the spectra for both materials show a very large decrease in the intensity of absorption bands for the range 1264–1227 cm^−1^, which results from the asymmetric vibration of C-O-C groups [88]. The absorption band derived from the symmetric vibration of C-O-C groups [88] at about 1174 cm^−1^ on the PBS spectrum also changed to a significant degree. After biodegradation, its maximum shifted to 1154 cm^−1^, which is due to the hydrolysis reaction of polymer chains, leading to the formation of alcohol (C-OH) groups [88]. As for the WB30 composite material, this change is less significant due to the presence of the biofiller with its structure containing -OH groups in polysaccharides. In addition to that, a new absorption band occurred in the spectra of the biodegraded PBS at 1327 cm^−1^, and the intensity of the band at 1330 cm^−1^ increased significantly in the spectrum of WB30. This is most likely related to the formation of carboxyl groups through hydrolysis [47].

Considering the region of carbonyl group vibration [89] at about 1720 cm^−1^, it becomes apparent that the intensity of this band clearly decreased compared to the vibration bands of C-O groups at approx. 1154 cm^−1^. This proves that ester groups disappear during biodegradation and are replaced by alcohol and possibly carboxyl groups. Moreover, the disappearance of absorption bands at about 990 cm^−1^ and 865 cm^−1^, characteristic of C-COO stretching vibrations in ester [90], also proves that ester bonds are degraded during composting. Also, this biodegradation pattern is evidenced by the band at about 3300 cm^−1^ originating from the vibration of -OH groups [88]. The increased intensity of this band is more evident for the biocomposite samples than for PBS, as the biocomposites biodegrade to a much greater extent than PBS.

### 3.3. Molecular Mass

**Figure 3:**
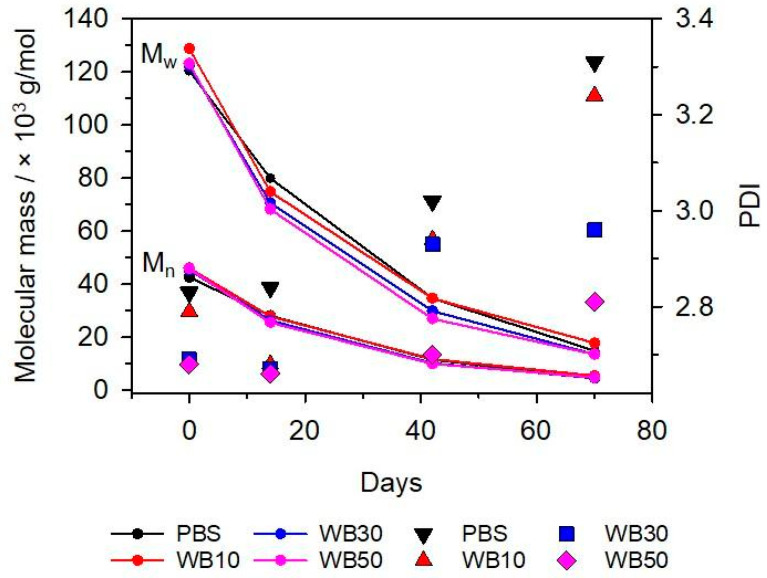
Average molecular weights (Mn, Mw; lines) and PDI (symbols) of PBS and biocomposite samples after composting.

Molecular mass changes in PBS after specified biodegradation times were assessed by gel permeation chromatography (GPC). Obtained chromatograms (Supplementary Figures S1–S4) were used to calculate the average molecular mass (M_n_), the weight average molecular mass (M_w_) and polydispersity index (PDI), as shown in Figure 3. It can be observed that the PBS and biocomposite samples before composting have similar values of M_n_ (~45,000 g/mol) and M_w_ (~125,000 g/mol), with PDI close to 2.7. When increasing the composting time, the M_n_ and M_w_ values decrease, whereas the PDI value increases. The rate of molecular mass decreasing with composting time is similar for all tested samples. These findings agree with the FTIR results. The FTIR analysis confirmed the breakdown of ester linkages during composting. This led to the disintegration of long PBS chains, primarily by the random chain scission mechanism [47,51], causing the observed decrease in M_n_ and M_w_. For the biocomposite samples, the hydrolysis of bran induced by the compost microorganisms also accelerated the rate of polymer chain scission due to greater access to the polymer matrix.

### 3.4. Morphology

**Figure 4:**
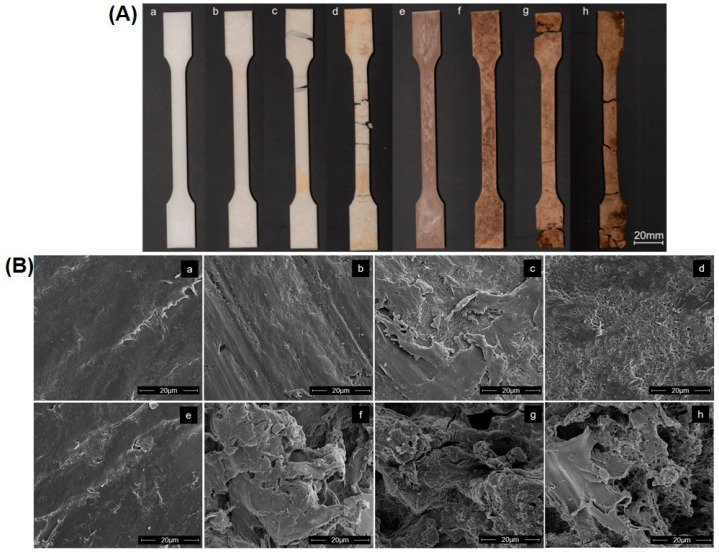
(**A**) Photographs of dog-bone-shaped specimens and (**B**) SEM images of PBS (**a**) before biodegradation and (**b**) after 14 days, (**c**) 42 days, (**d**) 70 days of biodegradation; WB30 (**e**) before biodegradation and (**f**) after 14 days, (**g**) 42 days, (**h**) 70 days of biodegradation.

Figure 4A shows the photographs of PBS and WB30 surface (colours mark different wheat bran contents [65]) before and after biodegradation in compost. Naked eye examination shows clear differences between the initial and composted samples. Although both PBS and WB30 disintegrated after 70 days, the WB30 samples show a greater degree of degradation. For better insight into morphological changes in the samples during composting, an SEM analysis was performed. SEM images of PBS and WB30 are shown in Figure 4B. The surface of PBS before composting is smooth, with no visible pores. In contrast, the surface of WB30 is rougher than that of pure PBS; small cracks and grooves are visible, resulting from the presence of bran particles. Since it is difficult to distinguish between individual bran particles, it can be concluded that the bran particles are homogeneously dispersed in the PBS matrix. During biodegradation, considerable modifications can be observed on the PBS and WB30 surfaces. For PBS, significant changes took place after 42 days (Figure 4B(c)), and after 70 days (Figure 4B(d)), a highly porous structure is visible, which suggests the decay of the polymer. On the other hand, the surface of WB30 shows substantial morphological changes after just 14 days. Besides pores, bigger holes are visible. Most probably, they were formed after the hydrolysis and decay of bran particles. When increasing the composting time, the surface of the sample becomes more porous, and deep cracks and holes appear (Figure 4B(g,h)). The SEM results clearly show that the biodegradation rate of the composite sample was higher than that of pure PBS. Similar SEM results related to the biodegradation of PBS with different biofillers are reported in the literature [84,89,91,92].

### 3.5. Differential Scanning Calorimetry

**Figure 5:**
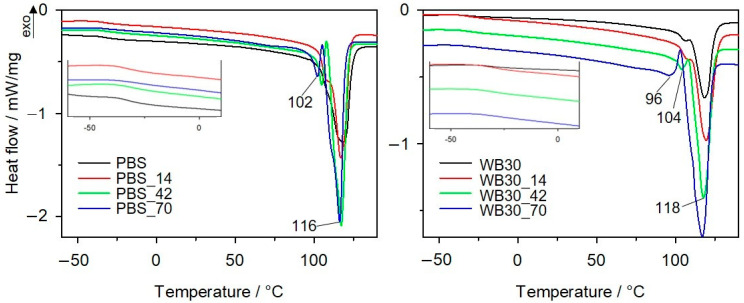
DSC thermograms (heating II cycle) with a *T_g_* region of PBS and WB30 for different biodegradation times.

DSC results of glass transition temperature (*T_g_*), crystallization temperature (*T_c_*), melting temperature (*T_m_*), melting enthalpy (∆*H_m_*) and the degree of crystallinity (*X_c_*) of the samples before biodegradation and after different composting times are listed in Table 1. DSC thermograms for heating II are given in Figure 5 and Supplementary Figure S5. PBS and its bran composites clearly differ in the degree of crystallinity before and after biodegradation. For all materials, *X_c_* increases with increasing biodegradation time. Considering the *X_c_* values calculated from Δ*H_m_*, the smallest changes occurred for pure PBS, for which *X_c_* increased by 16.7% after 70 days of composting. On the other hand, the bran-filled composites show a much higher increase in *X_c_*. It should be noted that for the calculation of post-biodegradation composite crystallinity, the real WB content was calculated based on the first mass loss from the TG curves. This mass loss comes from the thermal decomposition of WB [63]. Owing to the complexity of the chemical structure of the PBS/bran composites, it is difficult to directly determine from the FTIR spectra that the chemical structure of the bran-building substances changed during composting; nevertheless, the literature reports that these substances are susceptible to microorganisms in compost and undergo enzymatic hydrolysis [51,85]. The SEM analysis of the WB30 composite confirmed this observation. Moreover, an analysis of the FTIR spectra showed that the changes in the chemical structure of PBS occurred within the ester groups, while the GPC analysis confirmed the breakdown of polyester chains, leading to a decrease in the molar mass of PBS. The ability to form a crystalline phase increased with decreasing the molar mass of the polymer, which accounts for the crystallinity degree increase after composting. Additionally, the crystallinity degree increase observed for the PBS and composites can also be linked to the fact that the amorphous phase of the polymer is the first to biodegrade [84,93,94].

An analysis of glass transition temperature during biodegradation reveals a slightly downward trend. The lowest *T_g_* value was observed in the samples after 70 days of composting. This can be explained by a higher degree of crystallinity of these samples and a loss of the amorphous phase content [94]. It is also worth focusing on the melting peaks in the DSC curves. All composted materials show a bimodal melting peak, and therefore, two melting temperatures (*T_m_*_1_, *T_m_*_2_) are given in Table 1. For PBS and WB10, an additional peak at around 105 °C can clearly be distinguished after 42 and 70 days of composting, and after 14 days, a shoulder on the main melting peak is visible. The composites with higher bran contents, i.e., WB30 and WB50, show the first melting peak at about 106 °C after 14 and 42 days of biodegradation. On the other hand, after 70 days, a broad single melting peak with a shoulder is observed at around 120 °C. The presence of the two melting peaks in the thermograms indicates that two populations of crystallites of different sizes are present in the structure of these materials. The biodegradation process led to the breakdown of polymer chains by the chain scission mechanism and, consequently, to the reduction in their average molar masses. Accordingly, the polymer chains had greater mobility and could solidify into crystalline structures, which led to their higher crystallinity degree. Furthermore, the PDI value increase with composting time indicates that the PBS chains were characterized by a large size dispersion, which may have been responsible for the formation of a variety of crystallites, including weaker-formed ones with lower melting points. Similar observations were made for PBS composites with bran subjected to the accelerated aging test [65] or PBS composites with hemp fibres or hemp shives exposed to enzymatic hydrolysis and soil burial [94].

### 3.6. Thermal Resistance

**Figure 6:**
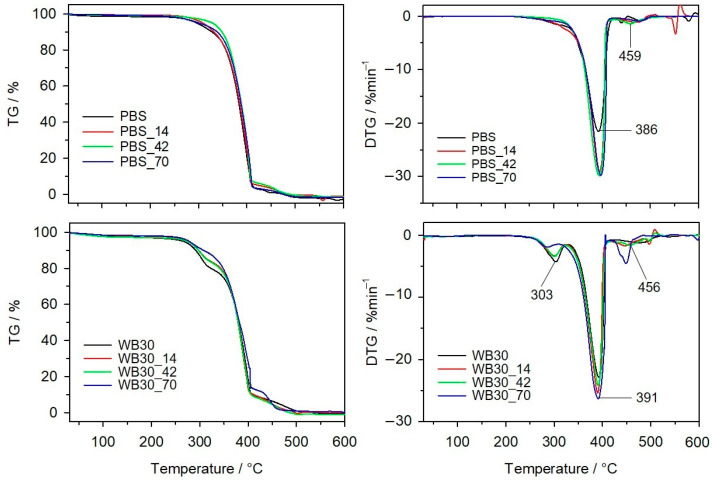
TG and DTG curves for PBS and WB30, before and after composting.

**Figure 7:**
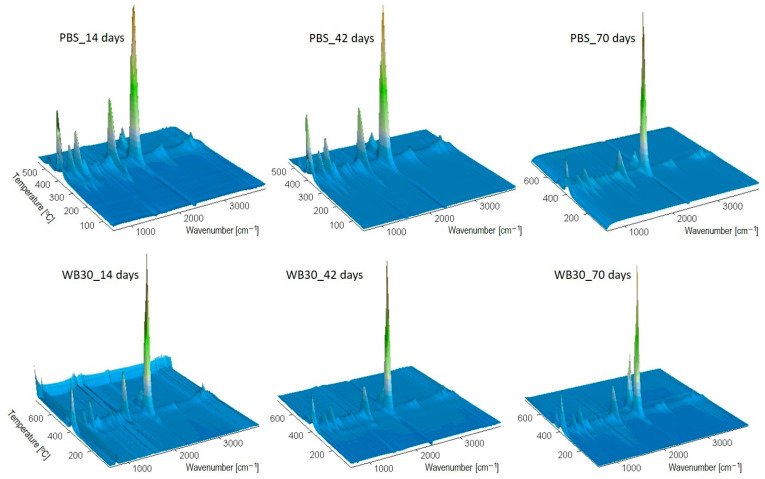
Three-dimensional FTIR diagrams showing gaseous degradation products of PBS and WB30 for different composting times.

The thermal resistance of PBS and its composites before biodegradation and after specified biodegradation times was tested under an oxidative atmosphere. Figure 6 shows the TG and DTG curves of the samples for different biodegradation times, while Table 2 lists the parameters describing the thermal resistance of the samples. The results clearly demonstrate that composting caused changes in the thermal resistance of the tested samples. The *T*_5%_ value corresponding to a 5% mass loss in the sample can be taken as the mass loss onset temperature. Thus, pure PBS undergoes thermal decomposition at about 308 °C before composting, and the mass loss temperature slightly decreases with the biodegradation time. The DTG curves before and after composting show that PBS undergoes a two-stage decomposition with the maximum at around 385 °C and 470 °C, but the decomposition rate is higher for the biodegraded PBS. However, an analysis of the 3D FTIR emission diagrams of the gaseous decomposition products for PBS_14 and PBS_42 (Figure 7) demonstrates clearly that the emission begins as early as around 290 °C. Previous studies on accelerated ageing [65] showed a similar thermal decomposition rate for aged PBS. Given the nature of the DSC curves and the GPC results obtained for composted PBS, it can be concluded that the degradation of short polymer chains formed by hydrolysis occurs at this stage. The FTIR spectrum of gaseous PBS decomposition products at about 290 °C (Figure 7) shows the absorption bands at 909 cm^−1^ (vibration of -COOH), 1053 cm^−1^ (vibration of -C-O-C-), 1207 cm^−1^ (vibration of -C-OH), 1818 cm^−1^ (vibration of C=O) and 2800–2900 cm^−1^ (vibration of -CH_3_, -CH_2_-) [88]. These absorption bands are characteristic of succinic acid and butane-1,4-diol [95], and their presence confirms that the thermal degradation of PBS starts with the hydrolysis of ester groups. This process is accompanied by decarboxylation and oxidation since the absorption bands derived from carbon dioxide (at 2359–2310 cm^−1^ and 669 cm^−1^) [95] and water (broad bands at approx. 4000–3500 cm^−1^ and 1800–1300 cm^−1^) [95] are present in the spectrum. In the case of PBS_70, which showed a significant degree of biodegradation, no gas emission was observed at 290 °C. Its structure contains a high proportion of crystalline phase, which is thermally more stable than the amorphous phase. In the next degradation step at approx. 390 °C, the absorption bands derived from succinic acid and butane-1,4-diol are still present in the FTIR spectrum, but the emissions of oxidation-induced gaseous products, i.e., carbon dioxide and water, can also be observed.

Regarding the biocomposite samples, their *T*_5%_ values increase with the biodegradation time, which is particularly evident for the WB50 sample containing the highest biofiller content. Three stages of thermal degradation of the samples can be distinguished before and after composting. The first stage primarily involves thermal degradation of the lignocellulosic bran-forming substances. The biodegradation process induced visible changes in the mass loss percentage Δ*T*_m1_ and decreased the *T_max_*_1_ value. Bran is susceptible to the microorganisms present in the compost and is the first to undergo enzymatic hydrolysis [47,85]; therefore, its percentage in the composite decreases. This results in a decrease in the Δ*T*_m1_ value calculated from the TG curves. In this stage, the FTIR spectra of gaseous decomposition products (Figure 7) are dominated by the absorption bands from carbon dioxide and water [95]. PBS decomposes at the second stage of thermal decomposition, reaching the maximum rate at *T_max_*_2_ of about 390 °C. An analysis of the absorption bands in the FTIR spectra confirms the emission of succinic acid, butane-1,4-diol, carbon dioxide and water, as was observed for pure PBS [95].

The final stage of decomposition in the materials composted for 42 and 70 days reaches its maximum at a temperature lower than that used for the starting materials and those biodegraded for 14 days, which is particularly evident for the biocomposite samples with a higher bran content. In this stage of decomposition, oxidation processes take place in both PBS and bran-based composites and are accompanied by the emission of dioxide and water.

## 4. Conclusions

The results of this study confirmed that the composting (biodegradation) time and the biofiller (bran) content had a significant impact on the structural and thermal properties as well as thermal resistance and percentage mass loss of the analysed composite samples. Conducted under industrial-like composting conditions, the biodegradation of pure PBS resulted in only about 5% mass loss, even after 70 days. The addition of bran to the material significantly accelerated its biodegradation, and the mass loss after 70 days exceeded 60% with 30% wt. and 50% wt. bran contents. The increased bran content in the composite also led to a high mass loss of the biodegraded composite and shortened the biodegradation time. The FTIR analysis showed changes in the chemical structure of PBS and biocomposite samples. Enzymatic hydrolysis resulted in the breakdown of ester bonds, forming the polymer chains. This finding was also confirmed by the average molar masses of the composted PBS samples. Both M_n_ and M_w_ values dropped significantly with the biodegradation time and were accompanied by an increase in PDI. The hydrolysis of ester bonds led to shortening polymer chains and increasing the crystallinity degree of the composted materials. The crystallinity degree increase resulted in the formation of numerous cracks in the injection mouldings due to a smaller volume of the crystalline phase than the amorphous phase and consequently gave rise to the formation of internal stresses. The thermogravimetric analysis of the biocomposite samples confirmed that the lignocellulosic material was the first to undergo biodegradation, which resulted in the improved thermal resistance of the samples.

## Figures and Tables

**Table 1:** DSC results for PBS and biocomposites before and after composting.

Sample	Cooling	Heating II			
	*T_c_*	*T_g_*	*T_m_*_1_/*T_m_*_2_	Δ*H_m_*	*X_c_*
	(°C)	(°C)	(°C)	(J/g)	(%)
PBS	86.2	−31.7	118	66.7	60.5
PBS_14	88.2	−32.5	107/117	70.9	64.3
PBS_42	86.4	−32.8	105/117	84.8	76.9
PBS_70	84.3	−34.5	102/116	85.2	77.2
WB10	86.3	−33.0	107/116	55.3	55.7
WB10_14	86.4	−33.1	107/119	67.5	67.6
WB10_42	87.9	−33.9	107/117	82.8	82.5
WB10_70	88.3	−34.1	104/115	85.2	83.1
WB30	79.6	−32	107/118	38.8	50.3
WB30_14	83.9	−32.8	106/119	47.6	57.5
WB30_42	83.7	−33.4	104/118	62.3	74.3
WB30_70	79.3	−35.4	96/117	93.5	96.3
WB50	83.3	−31.4	106/117	30.6	54.5
WB50_14	84.1	−31.9	108/121	44.3	73
WB50_42	83.5	−32.6	107/119	60.8	76.6
WB50_70	79.6	−33.6	103/120	68.6	79.7

**Table 2:** Thermal resistance of PBS and biocomposites before and after composting, based on data from thermogravimetric (TG) and derivative thermogravimetric (DTG) curves.

Sample	*T*_5*%*_	*T*_50*%*_	*T_max_*_1_	Δ*m*_1_	*T_max_*_2_	Δ*m*_2_	*T_max_*_3_	Δ*m*_3_
	(°C)	(°C)	(°C)	(%)	(°C)	(%)	(°C)	(%)
bran	201	303	296	68.0	-	-	459	29.7
PBS	308	393	-	-	386	97.9	463	2.1
PBS_14	306	395	-	-	385	94.5	471	5.5
PBS_42	305	395	-	-	388	95.3	459	4.7
PBS_70	308	398	-	-	388	96.4	475	3.6
WB10	299	383	305	8.5	387	84.3	478	7.2
WB10_14	312	387	304	8.1	395	87.8	468	6.2
WB10_42	298	385	303	7.5	394	85.9	479	5.9
WB10_70	296	381	286	6.3	391	82.5	468	7.4
WB30	274	381	303	19.8	391	71.1	476	9.1
WB30_14	276	379	301	16.8	390	73.6	446	9.6
WB30_42	277	379	300	16.1	391	74.2	456	9
WB30_70	279	382	297	13.2	391	73.9	449	12.9
WB50	231	372	300	32.4	386	53.5	462	14.1
WB50_14	233	372	297	29.5	386	57.6	443	12.9
WB50_42	265	377	288	18.2	386	66.9	417	14.9
WB50_70	271	371	285	14.1	375	66	410	19.9

## Data Availability

The data presented in this study are available on request from the corresponding author.

## Supplementary Materials

The following supporting information can be downloaded at: https://www.mdpi.com/article/10.3390/ma16216843/s1, Figure S1: GPC chromatograms obtained for PBS before and after the specified periods of composting; Figure S2: GPC chromatograms obtained for WB10 before and after the specified periods of composting; Figure S3: GPC chromatograms obtained for WB30 before and after the specified periods of composting; Figure S4: GPC chromatograms obtained for WB50 before and after the specified periods of composting; Figure S5: DSC thermograms (II heating cycle) of WB10 and WB50 biocomposites, obtained after a specified periods of biodegradation; Figure S6: TG and DTG curves for PBS and WB30 obtained before and after composting.
