# Supplementary material for: Study on the Biodegradation of Poly(Butylene Succinate)/Wheat Bran Biocomposites
Source: Materials (Basel). 2023 Oct 25;16(21):6843. doi: 10.3390/ma16216843 (PMC10647723; doi:10.3390/ma16216843)
Supplement: Supplementary file 1 [file materials-16-06843-s001.zip › materials-2659951-supplementary.pdf]

Study on the Biodegradation of Poly(Butylene Succinate)/ Wheat Bran Biocomposites

Supplementary data

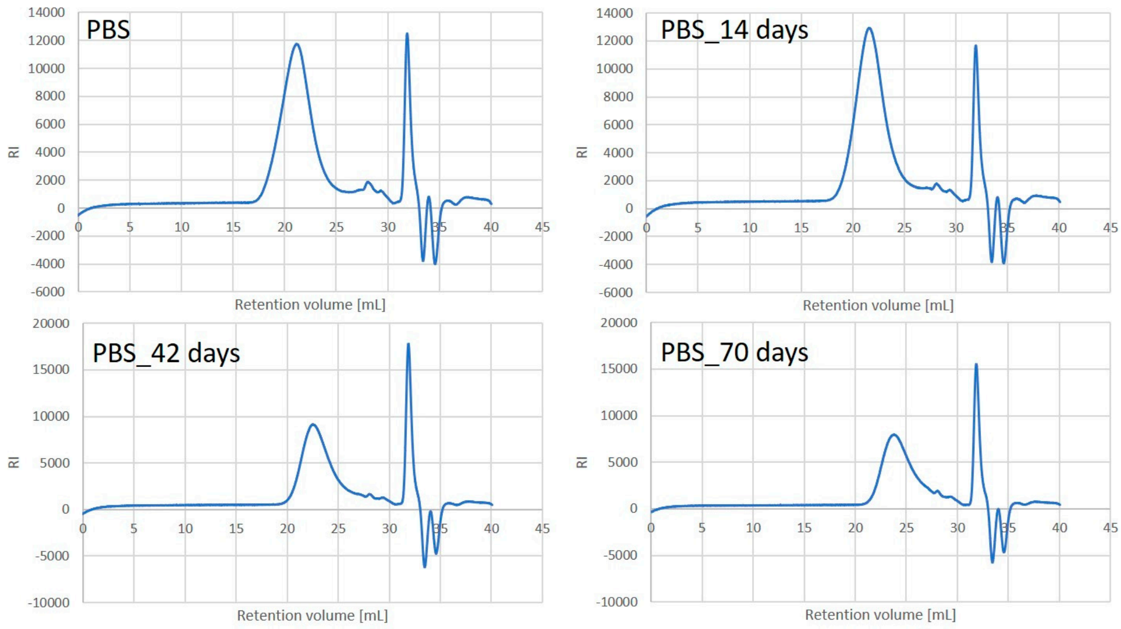

Figure S1. GPC chromatograms obtained for PBS before and after the specified periods of composting.

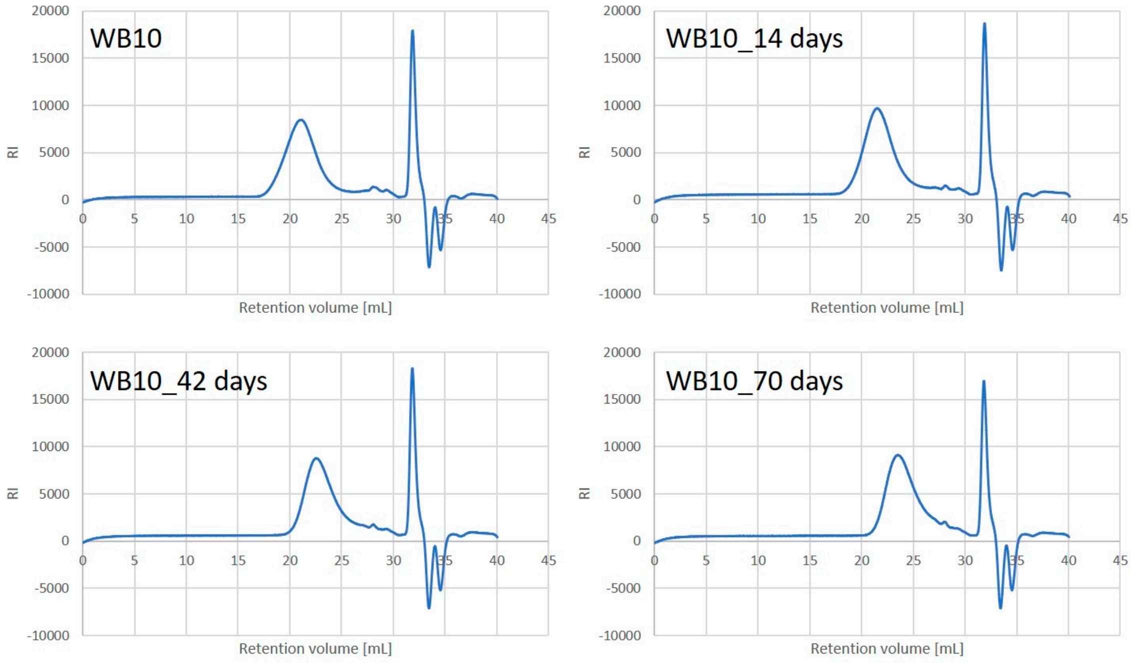

Figure S2. GPC chromatograms obtained for WB10 before and after the specified periods of composting.

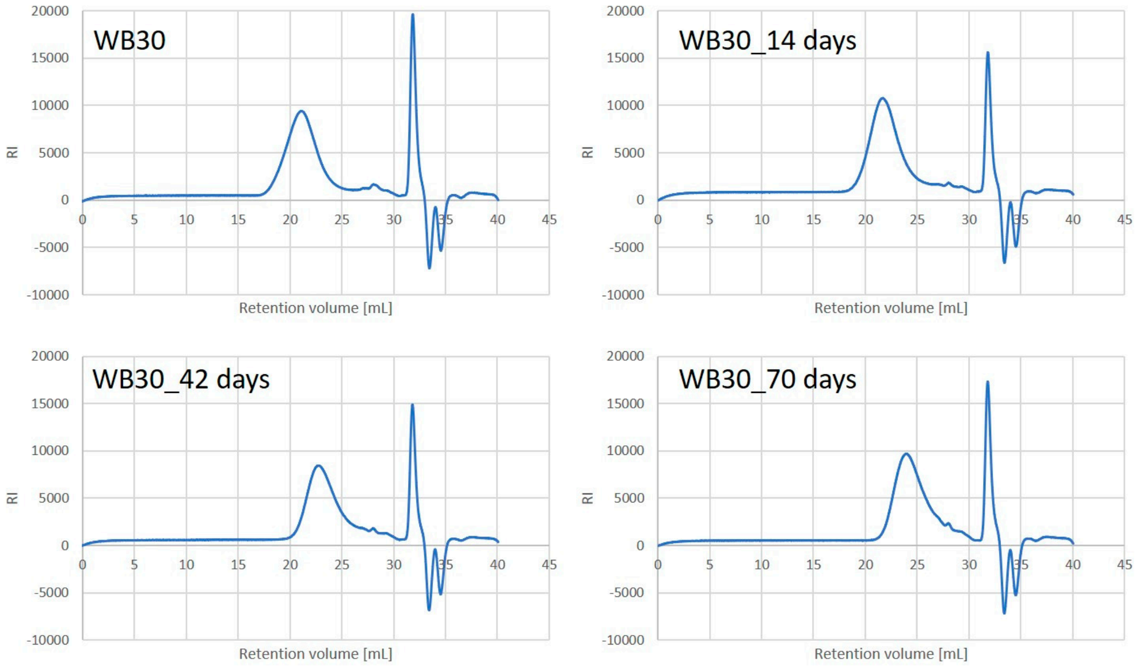

Figure S3. GPC chromatograms obtained for WB30 before and after the specified periods of composting.

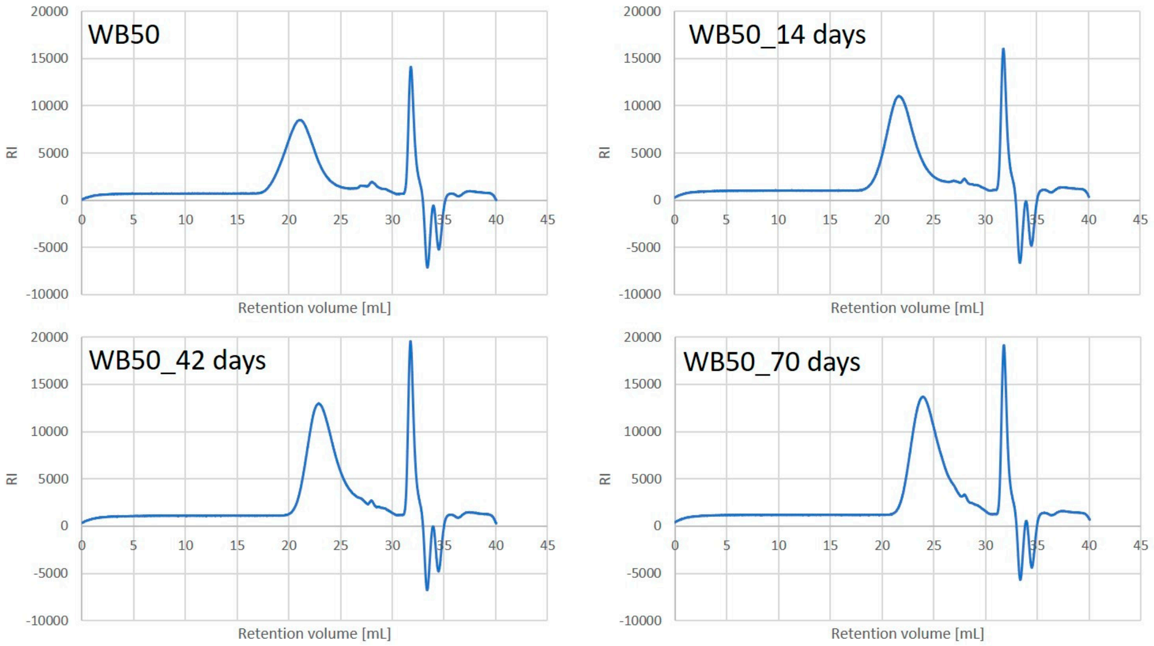

Figure S4. GPC chromatograms obtained for WB50 before and after the specified periods of composting.

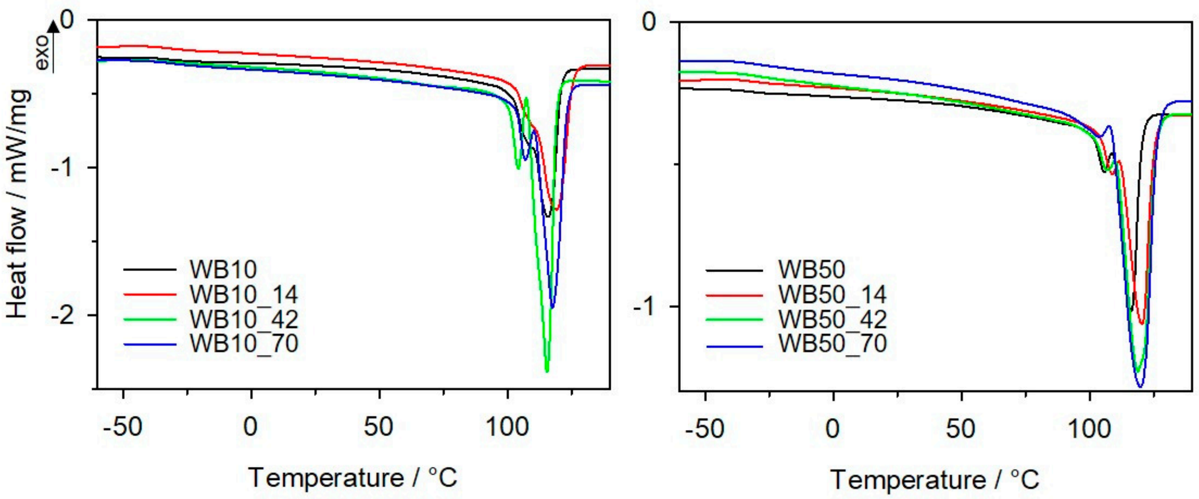

Figure S5. DSC thermograms (II heating cycle) of WB10 and WB50 biocomposites, obtained after a specified periods of biodegradation.

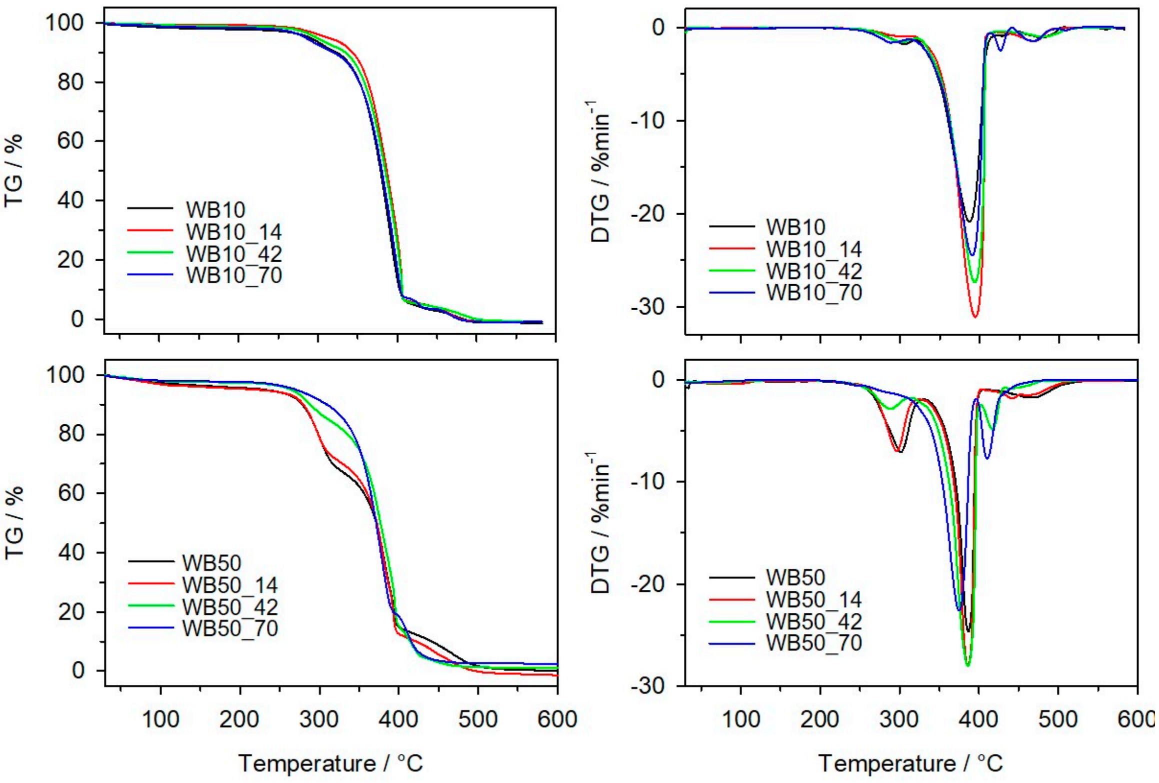

Figure S6. TG and DTG curves for PBS and WB30 obtained before and after composting.
